# Human retinal pigment epithelial cells express the long pentraxin PTX3

**Published:** 2013-02-06

**Authors:** Je Moon Woo, Min-Young Kwon, Da-Yong Shin, Young-Ho Kang, Narae Hwang, Su Wol Chung

## Abstract

**Purpose:**

To determine whether the long pentraxin 3 (PTX3) is expressed in human retinal pigment epithelial cells and is induced by inflammatory cytokines, interleukin-1 beta (IL-1β), tumor necrosis factor-alpha (TNF-α), and interferon-gamma (IFN-γ), expression of PTX3 was investigated in the human retinal pigment epithelial cell line, ARPE-19 cells.

**Methods:**

In ARPE-19 cells, we first analyzed PTX3 production in the presence or absence of inflammatory cytokines, IL-1β, TNF-α, and IFN-γ, dose- and time-dependently using enzyme-linked immunosorbent assay. Protein and mRNA expression of PTX3 was measured with western blotting analysis and real-time reverse transcription-polymerase chain reaction. Specific inhibitors were used to determine the signaling pathways of inflammatory cytokine-induced PTX3 expression.

**Results:**

In this study, production of PTX3 was induced by IL-1β and TNF-α dose- and time-dependently, but not by IFN-γ in ARPE-19 cells. Protein and mRNA expression of PTX3 was significantly upregulated in the presence of IL-1β and TNF-α. Furthermore, pretreatment with extracellular signal-regulated kinase1/2 and nuclear factor kappa-light-chain-enhancer of activated B cells specific inhibitor abolished IL-1β and TNF-α-induced PTX3 production, but the other inhibitors had no effect.

**Conclusions:**

These results suggested that human retinal pigment epithelial cells may be a major source of PTX3 production in the presence of proinflammatory cytokines, IL-1β and TNF-α, and could be an important mediator for host defense and inflammatory response in the retina. The importance of the mitogen-activated protein kinase/extracellular signal-regulated kinase1/2 and nuclear factor kappa-light-chain-enhancer of activated B cells pathways for regulated PTX3 expression may be a potential target for PTX3 regulation in the retina.

## Introduction

The retinal pigment epithelium (RPE) is a monolayer of pigmented cells situated between the neuroretina and the choroids and considered to be part of the retina [1]. The RPE contributes to the immune privileged status of the eye as part of the blood–retinal barrier and by the secretion of immunosuppressive factors inside the eye [2-5]. Physiologically, the RPE cell in maintaining photoreceptor excitability is the phagocytosis of shed photoreceptor outer segments [6-8]. The photoreceptor outer segments are digested, and essential substances such as retinoids are recycled and returned to the photoreceptors to rebuild the light-sensitive outer segments from the base of the photoreceptors. In addition, the RPE can secrete various growth factors that help maintain the structural integrity of the choriocapillaris endothelium and photoreceptors. Furthermore, the secretory activity of the RPE plays an important role in establishing the immune privilege of the eye by secreting immunosuppressive factors [9,10]. With these complex different functions, the RPE is essential for visual function. Failure of any one of these functions can lead to degeneration of the retina, loss of visual function, and blindness.

Immunologically, RPE cells play a pivotal role in the immune response. RPE cells orchestrate innate and adaptive immunity since they express Toll-like receptors (TLRs), complement components, major histocompatibility complex (MHC) class I and II molecules, and serve as antigen presenting cells [11-15]. RPE cells have TLRs, a family of cellular innate immunity molecules that sense molecular patterns associated with microbial pathogens. Moreover, both immune responses result in the production of cytokines, chemokines, and growth factors. Immune reactivity in RPE cells can be critically important in maintaining their functions as well as inflammation and infections.

Pentraxins are a superfamily of conserved proteins, characterized by a cyclic multimeric structure and a conserved C-terminal domain. Classic pentraxins, such as C-reactive protein and serum amyloid P, are acute-phase proteins that are rapidly activated in response to inflammation and play a role in immunity by regulating innate resistance to microbes and scavenging of cellular debris and components of extracellular matrix [16]. Pentraxin 3 (PTX3) is the prototypic long pentraxin, which shares similarity with the classic pentraxins in the C-terminal domain but has an unrelated N-terminal sequence [17,18]. Unlike classic pentraxins made in the liver, pentraxin 3 (PTX3; also called tumor necrosis factor-alpha [TNF-α]-stimulated gene 14) is rapidly produced and released by several cell types, endothelial cells, fibroblasts, and particularly mononuclear phagocytes, in response to either inflammatory or atheroprotective signals [19-21].

PTX3 levels are low in the sera and tissues of normal subjects but are rapidly increased in response to inflammatory stimulation with a wide range of diseases, including infectious, autoimmune, and degenerative disorders [22-24]. In particular, PTX3 acts as a soluble pathogen recognition receptor with an essential role in resistance against selected pathogens [21,24,25]. The regulated expression of this molecule in macrophages and dendritic cells suggests that PTX3 represents a mechanism of amplification of innate resistance against pathogens mainly acting locally at the site of infection and inflammation.

The RPE cell contributes to the immune privileged status of the eye as part of the blood–retinal barrier and by the secretion of immunosuppressive factors inside the eye. In this study, we examined the mechanisms by which early proinflammatory cytokines, interleukin-1 beta (IL-1β) and TNF-α, induce PTX3 production from human retinal pigment epithelial cells. We also characterized the signaling pathways triggered by IL-1β and TNF-α in PTX3 production.

## Methods

### Cell culture

ARPE-19 cells, human retinal pigmented epithelial cells, were purchased from the American Type Culture Collection (ATCC, Manassas, VA). ARPE-19 cells were cultured in a T-75 flask with Dulbecco’s modified Eagle’s medium (Invitrogen, Gibco, Carlsbad, CA) supplemented with 10% fetal bovine serum (FBS; Sigma, St Louis, MO) and 100 U⁄ml penicillin and streptomycin (Gibco-BRL, Gaithersburg, MD). During incubation, the culture medium was changed every 2 days. All cultures were maintained at 37 °C under 5% CO_2_ with 95% relative humidity.

### Antibodies and reagents

Protein kinase inhibitors (Enzo Life Science, Plymouth Meeting, PA), human TNF-α (R&D Systems, Inc., Minneapolis, MN), human IL-1β (Abcam, Cambridge, MA), and human INF-γ (PeproTech Inc., Rocky Hill, NJ) were used in the studies. Human PTX3 monoclonal antibody (Novus Biologicals, Littleton, CO) and β-actin polyclonal antibody (Santa Cruz Biotechnology, Inc., Santa Cruz, CA) were used for western blotting analysis. All signaling antibodies were purchased from Cell Signaling Technology, Inc. (Danvers, MA).

### RNA isolation and real-time reverse transcription-polymerase chain reaction

Total RNA from ARPE-19 cells was isolated TRIzol reagent (Invitrogen, Life technologies, Carlsbad, CA), and reverse transcription was performed using Moloney murine leukemia virus (M-MLV) reverse transcriptase (Invitrogen). cDNA was amplified with PCR, which was performed using the human *PTX3* specific primers and AccuPower PCR PreMix kit (Bioneer, Daejeon, South Korea). Expression of β-actin (*ACTB*) mRNA was used as the control. Primers were designed using Primer Express 1.5 software (Applied Biosystems, Carlsbad, CA) and synthesized from Bioneer Inc. (Daejeon, South Korea). The primer sequences were as follows: human *PTX3* forward primer, 5′-AAT GCA TCT CCT TGC GAT TC-3′; reverse primer, 5′-TGA AGT GCT TGT CCC ATT CC-3′ and *ACTB* forward primer, 5′-ATG GTG CGT GAC ATT AAG GAG AAG-3′; reverse primer, 5′-AGG AAG GAA GGC TGG AAG AGT G-3′. Amplification of cDNA started with 10 min at 95 °C, followed by 40 cycles of 15 s at 95 °C and 1 min at 59 °C. Real-time quantitative PCR for *PTX3* and *ACTB* was conducted using iQ SYBR Green Supermix (Bio-Rad, Hercules, CA).

### Western blot analysis

Western immunoblotting was performed as previously described [26]. Briefly, the cells were harvested using RIPA buffer (Sigma-Aldrich) with protease inhibitors (Roche Applied Science, Mannheim, Germany). Protein concentrations of cell lysates were determined using the Pierce BCA protein assay kit (Thermo Scientific, Rockford, IL). The samples were resolved with 12% sodium dodecyl sulfate–PAGE gels, and transferred to polyvinylidene difluoride membranes (Bio-Rad) overnight (120 mA). The transferred membranes were hybridized with various antibodies (diluted 1:1,000; Cell Signaling Technology, Inc.) overnight at 4 °C, followed by horseradish peroxidase-conjugated immunoglobulin G (HRP-conjugated IgG), and visualized with SuperSignal West Pico Chemiluminescent Substrate (Pierce, Rockford, IL).

### Enzyme-linked immunosorbent assay

The human PTX3 released into the culture medium was measured using an enzyme-linked immunosorbent assay (ELISA) kit (human PTX3/TSG-14) from R&D Systems, Inc. (Minneapolis, MN), following the manufacturer’s instructions. In brief, the ELISA plates (BD Biosciences, San Jose, CA) were coated with a monoclonal antihuman PTX3 antibody (2 μg/ml) in coating buffer (1% BSA in PBS (150 mM NaCl, 5 mM KCl, 5 mM Na_2_HPO_4_, 2 mM KH_2_PO_4_; pH 7.2–7.4) for overnight at room temperature. Then the plates were blocked with coating buffer for 2 h at room temperature, and incubated with either recombinant human PTX3 standards or the samples collected in quadruplicate (100 μl/well) for another 2 h. The plates were then incubated with a biotinylated human PTX3 antibody (150 ng/ml) for 2 h, and freshly diluted streptavidin-horse radish peroxide (HRP) for 20 min subsequently in the dark. After each step, the plates were washed three times with the washing buffer. The chromogen substrate tetramethylbenzidine (100 μl/well; eBioscience, Inc., San Diego, CA) was added and incubated for 5 min in the dark. The reaction was stopped by adding 2 N H_2_SO_4_ (50 μl/well), and the plates were read at 450 nm with an automatic ELISA reader (MERK SensIdent Scan, Helsinki, Finland).

### Statistical analysis

Data are represented as mean±standard deviation (SD). For comparisons between two groups, we used the Student two-tailed unpaired *t* test. For comparisons of timed series experiments, we performed Student paired *t* tests. Statistically significant differences were accepted at p<0.05.

## Results

**Figure 1:**
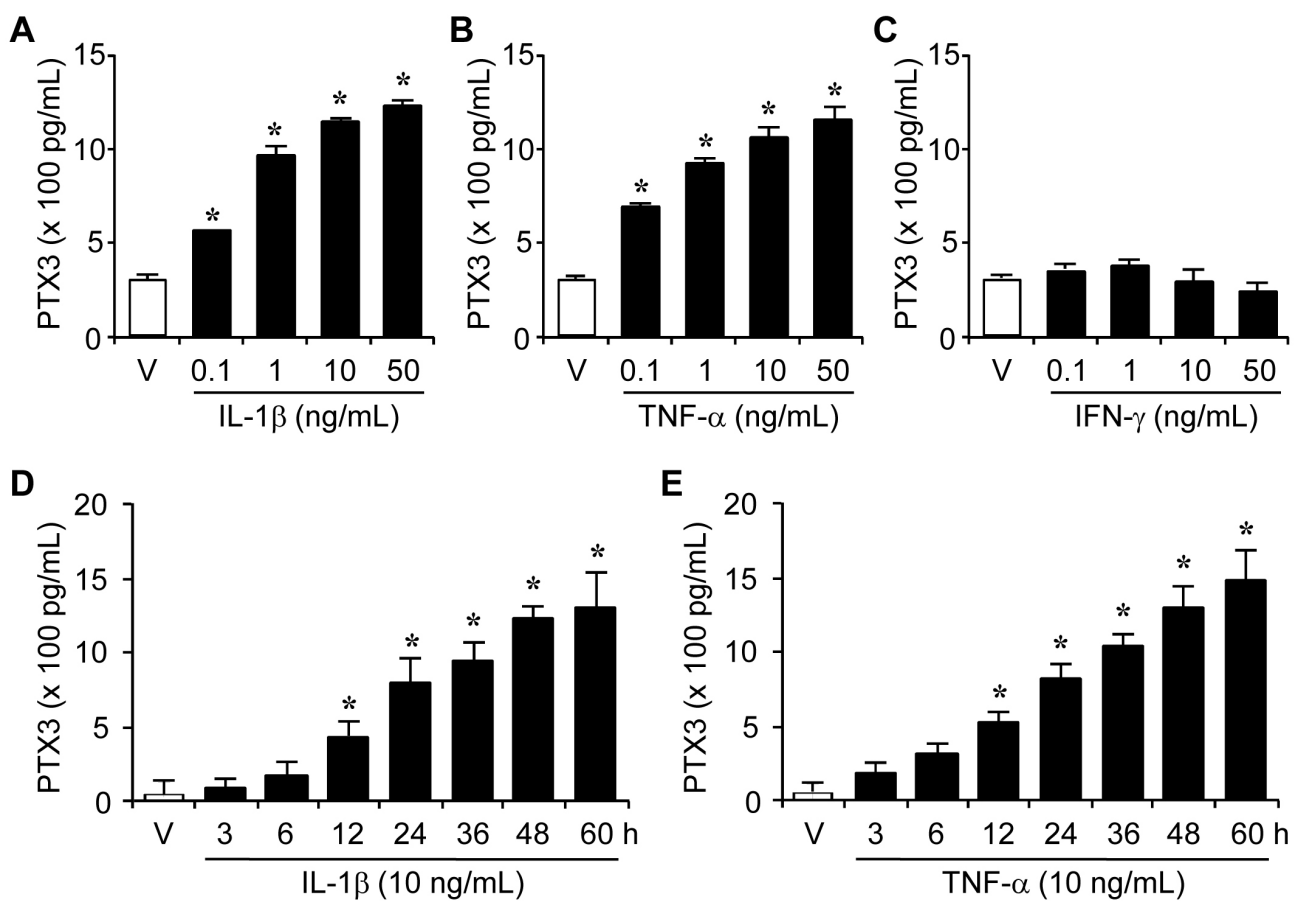
Production of pentraxin 3 (PTX3) by stimulation of inflammatory cytokines, interleukin 1 beta (IL-1β), and tumor necrosis factor alpha (TNF-α), in human RPE cells. ARPE-19 cells were stimulated with IL-1β (**A**), TNF-α (**B**), or IFN-γ (**C**) dose-dependently. After 60 h, supernatants were harvested and assessed for PTX3 production. ARPE-19 cells were incubated for various time periods in the absence or presence of IL-1β (**D**) and TNF-α (**E**). Supernatants were harvested and measured for PTX3 production at the indicated time. Values are mean±SD. Results are representative for three independent experiments. *p<0.05 versus vehicle.

The important roles of PTX3 have been suggested in the complement-mediated clearance of apoptotic cells and an amplification mechanism of innate resistance; however, expression of PTX3 in retinal pigment epithelia cells has not been reported. To determine whether PTX3 is produced in human retinal pigment epithelial cells, ARPE-19 cells (the human retinal pigment epithelial cell line) were stimulated with vehicle and proinflammatory cytokines, PTX3′s well known inducer, IL-1β (0, 0.1, 1, 10, and 50 ng/ml), TNF-α (0, 0.1, 1, 10, and 50 ng/ml), or IFN-γ (0, 1, 10, and 50 ng/ml). PTX3 production was measured with ELISA from supernatants after 60 h administration of vehicle, IL-1β, TNF-α, or IFN-γ. IL-1β and TNF-α induced PTX3 production dose-dependently (Figure 1A,B), but IFN-γ did not (Figure 1C). Furthermore, ARPE-19 cells were treated with IL-1β (10 ng/ml) or TNF-α (10 ng/ml) and harvested supernatants at 3, 6, 12, 24, 36, 48, and 60 h after cell treatment. PTX3 production began to increase by 6 h, and a more striking increase in PTX3 was evident after 12 h of IL-1β or TNF-α treatment (Figure 1D,E, respectively). The stimulating effects of IL-1β and TNF-α on PTX3 production were markedly enhanced up to 60 h. These data suggest that proinflammatory cytokines, IL-1β and TNF-α, could stimulate the production of PTX3 in human retinal pigment epithelia cells dose- and time-dependently.

**Figure 2:**
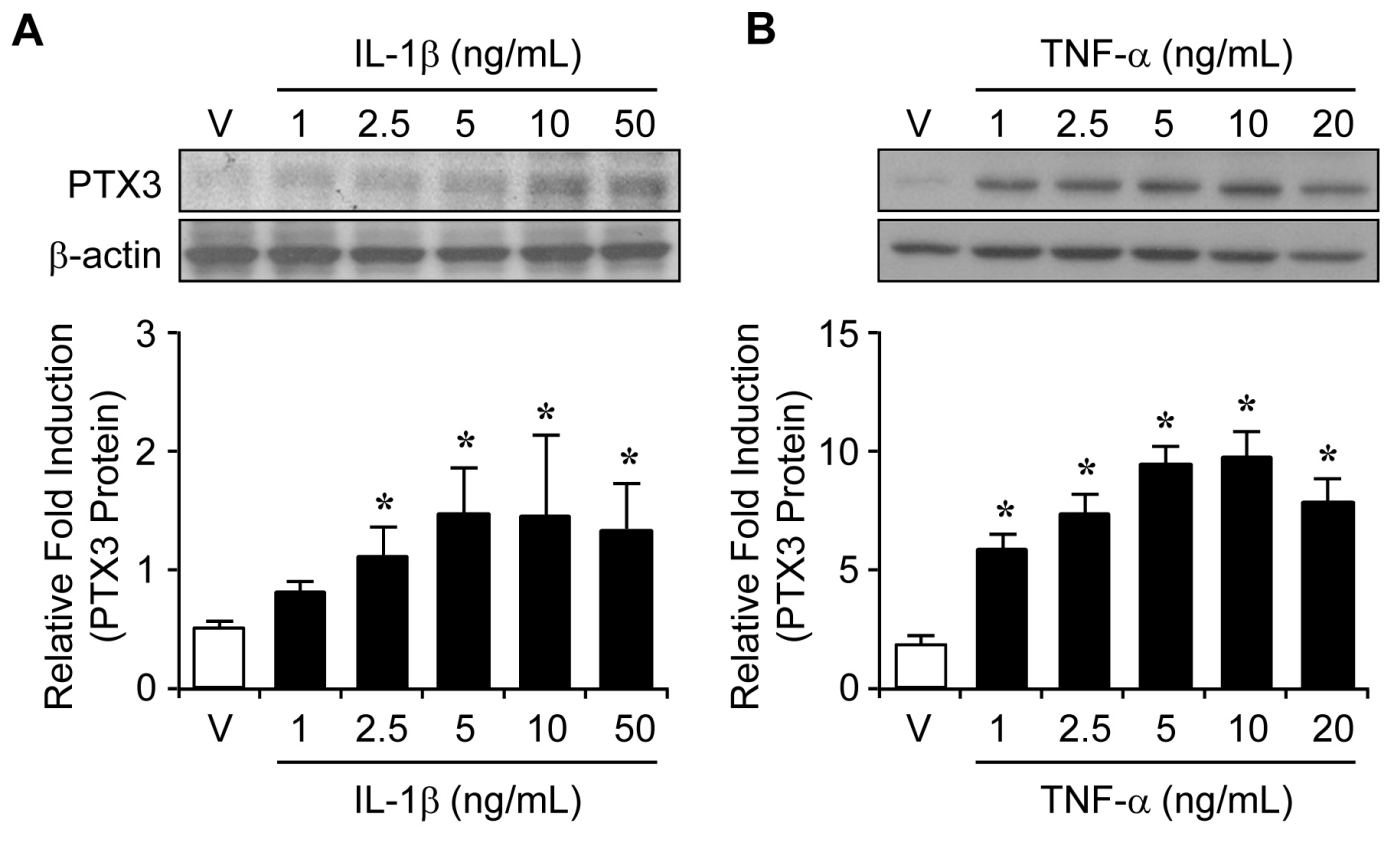
Induction of pentraxin 3 (PTX3) protein expression by interleukin 1 beta (IL-1β) and tumor necrosis factor alpha (TNF-α) in human RPE cells. The expression of PTX3 protein levels was assayed in the absence or presence of IL-1β (**A**) and TNF-α (**B**) in ARPE-19 cells. After 24 h treatment of IL-1β or TNF-α, PTX3 protein levels were analyzed with western blotting analysis with a human anti-PTX3 polyclonal antibody. These experiments were performed at three independent times, and the fold change in protein levels was quantitated as signal intensity corrected for loading in vehicle cells (white bars) or cells exposed to IL-1β (black bars) or TNF-α (black bars). Values are mean±SD. Results are representative for three independent experiments. *p<0.05 versus vehicle.

**Figure 3:**
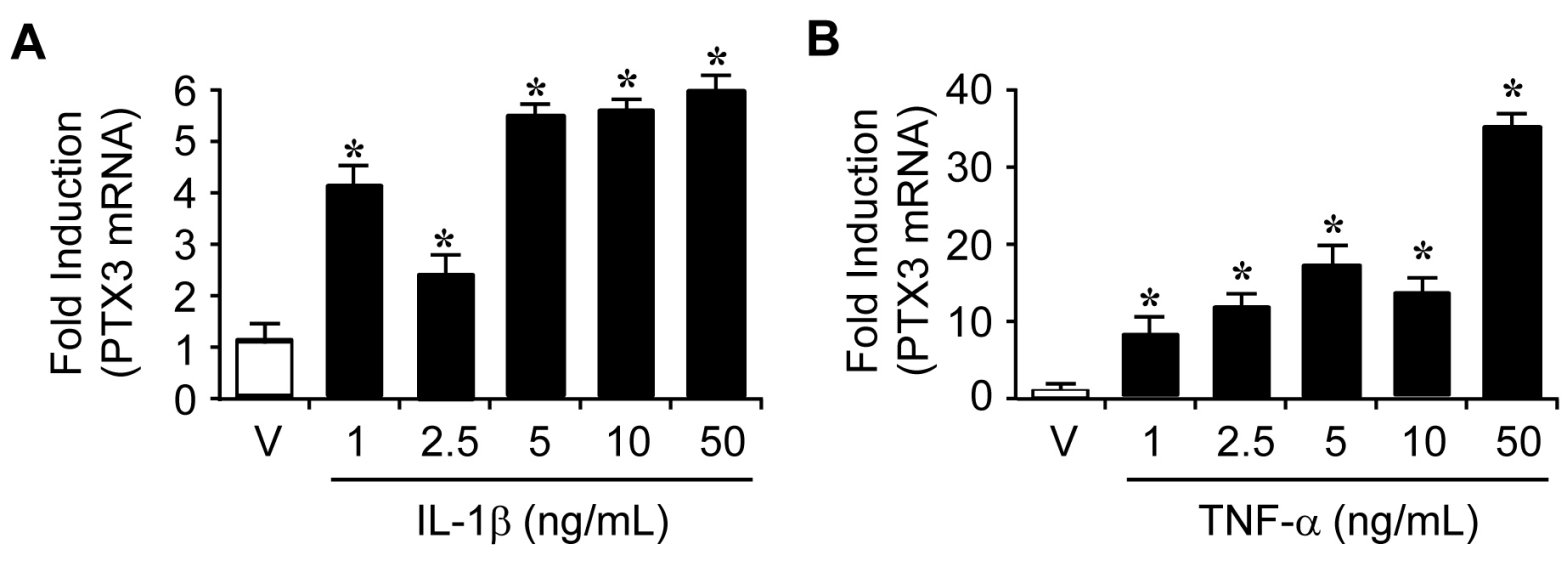
Induction of pentraxin 3 (PTX3) mRNA expression by interleukin 1 beta (IL-1β) and tumor necrosis factor alpha (TNF-α) in human RPE cells. Total RNA was extracted from ARPE-19 cells after various doses of IL-1β (**A**) or TNF-α (**B**) were administered after 24 h. The expression of the *PTX3* mRNA levels was assessed with real-time RT–PCR analysis. These experiments were performed at three independent times. Expression levels of *PTX3* is divided by expression of the control gene, *ACTB*, and shown as a ratio of *PTX3*/*ACTB*. *p<0.05 upregulation in IL-1β (**A**) or TNF-α (**B**) treatment versus no IL-1β or TNF-α. For *PTX3* mRNA, expression was normalized by an internal control gene, β -actin, and shown as a fold increase. For the real-time PCR experiments, values are presented as mean±SD, n=3.

To determine the effects of IL-1β and TNF-α on the protein and mRNA levels of PTX3 in ARPE-19 cells, protein and total RNA were isolated from ARPE-19 cells 24 h after increasing doses of IL-1β (0, 1, 2.5, 5, 10, and 50 ng/ml) or TNF-α (0, 1, 2.5, 5, 10, and 20 ng/ml) were administered. The PTX3 protein levels were increased from 2.5 ng/ml of IL-1β dose-dependently (Figure 2A). However, TNF-α enhanced the protein levels of PTX3 in a lower concentration (1 ng/ml) than IL-1β and maintained up to 10 ng/ml (Figure 2B). At the same time, administration of IL-1β or TNF-α led to similar enhancement of *PTX3* mRNA expression (Figure 3A,B, respectively). These results suggest that IL-1β and TNF-α increased PTX3 production at the translation and transcription levels.

**Figure 4:**
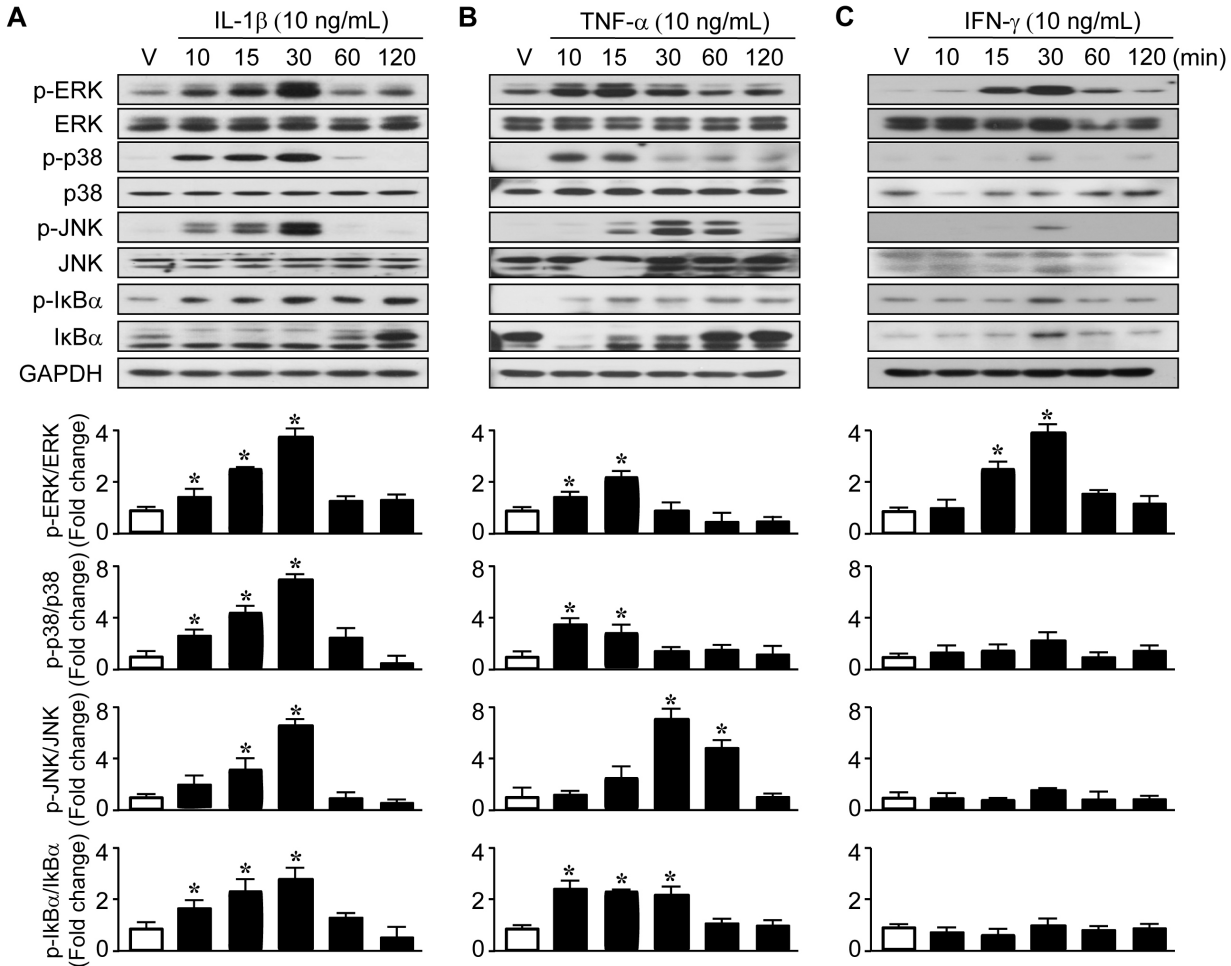
Induction of the signaling pathway in the presence of interleukin 1 beta (IL-1β), tumor necrosis factor alpha (TNF-α), and interferon gamma (IFN-γ). In ARPE-19 cells, cell lysates were analyzed with western blot for phosphorylated MAP kinase signaling protein (extracellular-signal-regulated kinases [ERK1/2], p38, and c-Jun N-terminal kinase [JNK]) and IκB and total mitogen activating protein (MAP) kinase signaling protein and IκB after IL-1β (10 ng/ml; **A**), TNF-α (10 ng/ml; **B**), or IFN-γ (10 ng/ml; **C**) administration. Glyceraldehyde 3-phosphate dehydrogenase (GAPDH) was used as a loading control. Immunoblot analyses of ERK1/2, p-ERK1/2, p38, p-p38, JNK, p-JNK, IκB, and p-IκB and densitometric quantification (bottom) with or without IL-1β, TNF-α, or IFN-γ, as indicated. Results are representative for three independent experiments. *p<0.05 versus vehicle.

**Figure 5:**
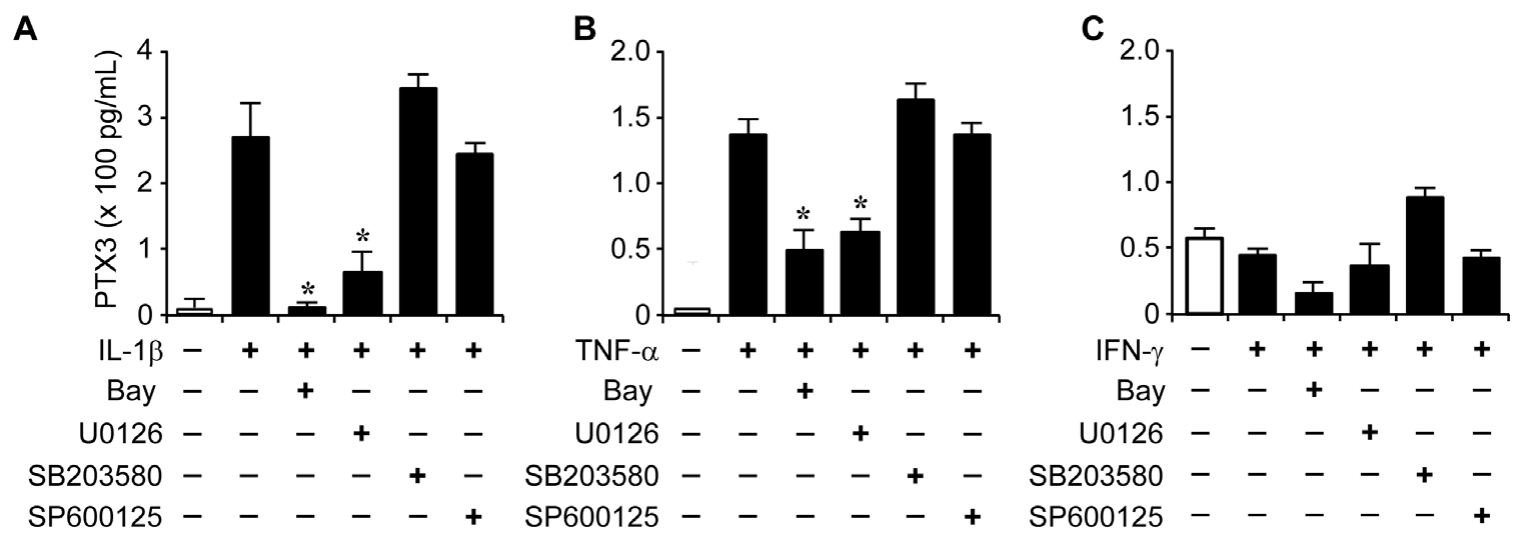
Downregulation of pentraxin 3 (PTX3) production by extracellular-signal-regulated kinases (ERK) 1/2 and/or nuclear factor kappa-light-chain-enhancer of activated B cells (NF-kB) signaling inhibitors. ARPE-19 cells were stimulated with interleukin 1 beta (IL-1β; **A**), tumor necrosis factor alpha (TNF-α; **B**), or interferon gamma (IFN-γ; **C**) in the absence or presence of mitogen activating protein (MAP) kinase and NF-κB signaling inhibitors. After 60 h, supernatants were harvested and assessed for PTX3 production. Results are shown as means±SD of three independent experiments. *p<0.05 versus vehicle.

To identify the signaling molecules involved in regulating PTX3 by IL-1β and TNF-α, we isolated protein from ARPE-19 cells after IL-1β (10 ng/ml), TNF-α (10 ng/ml), or IFN-γ (10 ng/ml) was administered. IL-1β and TNF-α did not have a significant effect on overall unphosphorylated p38, extracellular-signal-regulated kinases (ERK), or c-Jun N-terminal kinase (JNK). The phosphorylation time and intensity of signaling molecules were slightly different by IL-1β or TNF-α administration; however, phosphorylated p38, ERK, JNK, or IκB was increased by IL-1β or TNF-α (Figure 4A,B, respectively). Though phosphorylation of ERK was increased by IFN-γ, phosphorylation of p38, JNK, and IκB was weak (Figure 4C). We next assessed which signaling pathway(s) were responsible for stimulating PTX3 production by IL-1β, TNF-α, and IFN-γ. We used specific inhibitors of MAPK and NF-κB, U0126 (MEK1/2 inhibitor), SB203580 (p38 MAP kinase inhibitor), SP600125 (JNK MAP kinase inhibitor), and Bay 11–7085 (NF-κB inhibitor), respectively. The ARPE-19 cells were treated with U0126 (10 μM), SB203580 (10 μM), SP600125 (10 μM), and Bay 11–7085 (10 μM), in the presence of IL-1β, TNF-α, or IFN-γ, and supernatants were harvested 60 h after administration. U0126 and Bay 11–7085 blocked the stimulation of PTX3 production by IL-1β and TNF-α (Figure 5A,B, respectively). However, IFN-γ had no effect on PTX3 production in the presence of signaling inhibitors (Figure 5C). These data suggest that the ERK and NF-κB signaling pathways increase PTX3 production in the presence of IL-1β and TNF-α in human retinal pigment epithelial cells.

## Discussion

RPE cells absorb the light energy focused by the lens on the retina and contribute to maintaining photoreceptor excitability. Damage to any of the intricate structures of the retina can lead to visual impairment and blindness [2,3]. First, immune reactivity in the retina can be critically important in inflammation and infections, but regulation of this response is essential. The RPE cell, a unique retinal cell, displays several essential functions to support the health of the retina [1]. Immunologically, the RPE cell orchestrates innate and adaptive immunity. Especially, RPE cells express many different pattern recognition receptors (PRRs) such as Toll-like, nucleotide oligomerization domain-like, and scavenger receptors, and PRRs lead to a signal transduction cascade that generates a rapid and robust inflammatory response marked by cellular activation and production of several proinflammatory cytokines, chemokines, and adhesion molecules, including IL-6, IL-8, MCP-1, ICAM-1, and IFN-β [27-30]. A recent study showed that TLR polymorphisms have been associated with age-related macular degeneration (AMD) and investigation on the utility of small interfering RNA treatment for AMD identified that small interfering RNA was signaling through TLR3 [31,32].

Pentraxins are key components of the humoral arm of innate immunity, which include complements, collectins, and ficolins [16]. PTX3 is the first member of the long pentraxin subfamily, identified in the early 1990s as a new secreted protein rapidly induced by IL-1 in endothelial cells or by TNF in fibroblasts [17,18]. This molecule shares similarities with the short pentraxins but differs by the presence of a related long N-terminal domain as well as in the fields of gene organization, cellular source, and ligands that it recognizes [16-18]. PTX3 acts as a humoral innate immunity molecule, which is a non-redundant role in resisting selected pathogens by recognizing microbes, activating the complement, and facilitating pathogen recognition by phagocytes, and PTX3 is essential in female fertility [20,33-36]. PTX3 also acts as a scavenger of cell debris and as an immunosuppressant. PTX3 specifically binds to late apoptotic cells and subsequently inhibits their uptake by dendritic cells, thus acting as an additional tool for maintaining immune tolerance [37,38]. PTX3 binds to complement factor H, a complement regulator protein whose main function is to inhibit the activation of an alternative complement pathway. The polymorphic variant with the substitution of histidine for tyrosine at codon Y420H has been demonstrated to be strongly associated with AMD [39].

In this study, we found that human RPE cells, ARPE-19 cells, showed local production of PTX3, and expression and production of PTX3 were increased by IL-1β and TNF-α. These data suggested that PTX3 produced in RPE cells might have an important role in retinal injury against inflammation and infection. Moreover, PTX3 production and its role must be elucidated in the presence of pathological stimuli related to retinal degenerative diseases and other eye diseases.
